# Extension of the Lecture-term by the Medical Colleges

**Published:** 1848-04

**Authors:** 


EXTENSION OF THE LECTURE-TERM BY THE MEDICAL COLLEGES.

A late number of the Western Lancet, published at Cincinnati and edited by Professors Lawson and Harrison, contains a communication proposing that a Convention of delegates from the Western Schools shall meet at Cincinnati on the third Monday of the present month, "with full power to vote for a specific term for each winter session; also to decide on the expediency of abolishing the rule by which practitioners of four years' standing are permitted to become candidates for graduation, on attending one course of lectures, and also to abolish the credit system." We are glad to observe this movement on the part of the Western schools, and hope it will meet with their unanimous approval. None are so competent to decide on the propriety of extending the term, or the extent to which it should be carried, as those who have experience in the business of teaching. The other abuses proposed to be considered, are so glaring that the wonder is that any respectable school should countenance them.

